# Dynamics of Japanese Encephalitis Virus Transmission among Pigs in Northwest Bangladesh and the Potential Impact of Pig Vaccination

**DOI:** 10.1371/journal.pntd.0003166

**Published:** 2014-09-25

**Authors:** Salah Uddin Khan, Henrik Salje, A. Hannan, Md. Atiqul Islam, A. A. Mamun Bhuyan, Md. Ariful Islam, M. Ziaur Rahman, Nazmun Nahar, M. Jahangir Hossain, Stephen P. Luby, Emily S. Gurley

**Affiliations:** 1 Centre for Communicable Diseases, i,cddr,b, Dhaka, Bangladesh; 2 College of Public Health and Health Professions - Department of Environmental and Global Health, University of Florida, Gainesville, Florida, United States of America; 3 Department of Epidemiology, Johns Hopkins School of Public Health, Baltimore, Maryland, United States of America; 4 Department of Livestock Services, Ministry of Fisheries and Livestock, Dhaka, Bangladesh; 5 Global Disease Detection Branch, Division of Global Health Protection, Center for Global Health, Centers for Disease Control and Prevention, Atlanta, Georgia, United States of America; Emory University, United States of America

## Abstract

**Background:**

Japanese encephalitis (JE) virus infection can cause severe disease in humans, resulting in death or permanent neurologic deficits among survivors. Studies indicate that the incidence of JE is high in northwestern Bangladesh. Pigs are amplifying hosts for JE virus (JEV) and a potentially important source of virus in the environment. The objectives of this study were to describe the transmission dynamics of JEV among pigs in northwestern Bangladesh and estimate the potential impact of vaccination to reduce incidence among pigs.

**Methodology/Principal Findings:**

We conducted a comprehensive census of pigs in three JE endemic districts and tested a sample of them for evidence of previous JEV infection. We built a compartmental model to describe JEV transmission dynamics in this region and to estimate the potential impact of pig vaccination. We identified 11,364 pigs in the study area. Previous JEV infection was identified in 30% of pigs with no spatial differences in the proportion of pigs that were seropositive across the study area. We estimated that JEV infects 20% of susceptible pigs each year and the basic reproductive number among pigs was 1.2. The model suggest that vaccinating 50% of pigs each year resulted in an estimated 82% reduction in annual incidence in pigs.

**Conclusions/Significance:**

The widespread distribution of historic JEV infection in pigs suggests they may play an important role in virus transmission in this area. Future studies are required to understand the contribution of pig infections to JE risk in humans and the potential impact of pig vaccination on human disease.

## Author Summary

Japanese encephalitis (JE) virus infection can cause severe neurological disease in man. More JE cases are seen in northwestern districts in Bangladesh. Pigs are the most common amplifying host of the virus and can act as a potential environmental source. We conducted a comprehensive census of pigs in three JE endemic districts and tested a sample of them for evidence of previous JEV infection. We built a compartmental model to describe JEV transmission dynamics in this region and to estimate the potential impact of pig vaccination. We identified 11,364 pigs in our study area, mostly raised in backyards. About 30% of the pigs had evidence of previous JE virus infection. Our model suggests that vaccinating 50% of pigs each year resulted in an estimated 82% reduction in annual incidence in pigs. Pigs in northwestern Bangladesh may play a significant role in JE virus transmission. JE incidence may be substantially reduced through reasonable pig vaccination coverage.

## Introduction

Japanese encephalitis (JE) virus is an arthropod borne viral zoonosis that is endemic throughout eastern, south-eastern and southern Asian countries [1], [2]. JE virus (JEV) infection can cause irreversible damage to the central nervous system of humans, who serve as incidental ‘dead end’ hosts because they do not produce sufficient viremia to infect mosquitos [3]. Approximately, 60% of the world's population lives in JE endemic regions [4] and a 2011 review estimated that the annual incidence was 1.8/100,000 and 5.4/100,000 for children 0–14 years old in 24 JE endemic countries [5]. The majority of human infections are asymptomatic, and only a small ratio (1∶25 to 1∶1,000) develop clinical infections [6]. However, the prognosis for people who develop encephalitis is poor: approximately 25% die, and 30% to 60% of survivors suffer from neurological sequelae [7], [8].

JEV transmission is complex, involving numerous vertebrate and mosquito species, and is poorly understood in Bangladesh. Studies from other Asian countries show that *Culex* species are the primary vectors driving transmission; *Aedes* mosquitoes are also competent vectors but likely play only a minor role [9], [10]. Host species for the virus include ardeid wading birds and some domestic animals. In particular, pigs appear to play a major role in transmission cycles due to large-scale viral amplification and relatively high viral titers [11], promoting onward transmission [12], [13]. Several other domestic animal and bird species including cattle, goats, dog, ducks and chickens also become infected, but because they produce low level of viremia for a brief time, they are unlikely to play a significant role in transmission [14]–[17].

Hospital based acute meningoencephalitis surveillance in Bangladesh began in 2003 and identified that JEV infection was responsible for 6% of all encephalitis at surveillance hospitals [18]. Further work to characterize the burden of JE in 2009 estimated that the incidence was highest in the northwest part of the country, with 2.7 cases per 100,000 population per year [19], which is similar to its incidence in other JE endemic countries before the introduction of JE vaccine into national immunization programs [20], [21].

Although Bangladesh is a predominantly Muslim country, pigs are raised by some ethnic minority communities and in nomadic herds [22]. Understanding the distribution of pigs and the extent to which they get infected with JEV will help to define their role in the JEV transmission cycle. The objectives of this study were to describe the transmission dynamics of JEV among pigs in the northwestern part of Bangladesh, where human incidence is high, [19] and to estimate the potential impact of vaccinating pigs on JE incidence in the pig population.

## Methods

### Pig census and sampling

**Figure 1:**
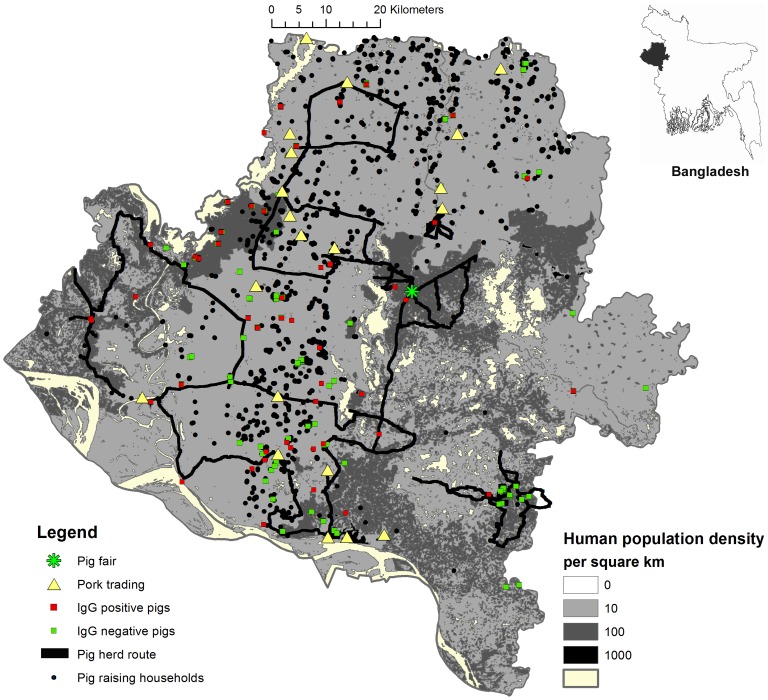
Map of pig-raising households, nomadic pig routes, pigs with antibodies to Japanese encephalitis virus, and human population density in Rajshahi, Nawabgonj, and Naogaon Districts, Bangladesh, 2009.

From May through September 2009 we used snowball sampling to identify all pig-raisers in Rajshahi, Nawabgonj, and Naogaon Districts and counted their pigs (Figure 1) [23]. Previous research suggested that pig-raising communities maintained close kinship networks and we relied on these networks to identify all pig-raising households in these districts [22]. We initially identified 10 pig-raisers who previously participated in a study of other diseases with us in 2007–2008 [22]. In addition, we collected a list of 100 pig-raisers identified through a local livestock office's preliminary survey to identify pig-raisers. Once a pig-raising household was identified, we asked them if they knew other pig-raisers in the study area. All identified pig-raising households were listed and visited and asked to identify other households. This process was continued until no additional pig-raisers were found. We then visited 20 randomly selected unions (smallest administrative unit consisting of multiple villages) where no pig-raising was reported and went to places where residents congregate, such as markets, tea stalls and shops, to ask if they knew about any pig-raising communities in those areas. The presence of pigs reported through these interviews was confirmed by household visits.

At each pig-raising household, we recorded the number of pigs, their age, sex, and location. We collected blood samples from pigs over six months of age for JE IgG antibody testing. We approached the first pig raising household in the village and sampled a pig after obtaining an informed consent. We continued the sampling in the nearest households until they sampled 10 pigs per village. If no more than 10 pig raising households were identified in a village, we collected samples from multiple pigs from a household. If there were less than 10 pigs in a village, we sampled all of the pigs. The sampling continued until we reached a sample size of 260.

We used the same snowball technique to identify any mobile pig herds in our study area. Regardless their health status, we recorded the number of pigs in each herd and collected blood from at least three pigs but no more than 10. Although we plan to sample 10 adult pigs per herd, the herders often provided less than that, or the herds did not have that many adult pigs for testing. The serum samples were tested by using a commercially available enzyme-linked immunosorbent assay (ELISA) according to manufacturer's protocol (GENTAUR BVBA – Genoprice, Belgium. http://www.genoprice.com/).

With guidance from herders from each herd, we recorded longitude and latitude of the grazing locations and travelling paths (from April 2008 and March 2009) of nine pig herds. These herds were particularly chosen for mapping because their grazing covered the majority of the nomadic pig herds feeding sites, and represented geographical variation of the feeding sites for the three districts.

We also conducted informal interviews with all nomadic pig herders to explore the commercial trade of pigs at markets and backyard raisers. We visited commercial pig traders at the markets and some backyard pig-raisers to verify the frequency and timing of the sale.

### Data analysis

We described the demographics of pigs in our study area and the proportion with evidence of historic exposure to JE. We mapped the locations of pig-raising households, nomadic pig-raising routes, and sampled pigs by JE IgG status.

#### Spatial heterogeneity in serostatus

To assess spatial heterogeneity of seropositive pigs, we first constructed intensity maps by placing a fine grid over the study area and estimating the probability of pigs within each grid segment being seropositive, using a Gaussian kernel with a bandwidth that minimized the cross-validated error [24]. We then used a resampling method to test the null hypothesis of no spatial heterogeneity in the proportion of seropositive pigs [25]. In the resampling method, we repeatedly randomly assigned serostatus of each tested pig, keeping the total number of seropositive and seronegative pigs the same. We then re-estimated the intensity plot and calculated the following test statistic:
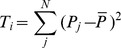



The test statistic for resample *i (T_i_)* represents the sum across all N grid cells of the squared difference between the estimated proportion of cases that were seropositive in grid cell *j* (*p_j_*) and the overall proportion of cases that were seropositive from all samples 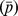. We repeated this resampling and test statistic calculation 499 times. Greater values of *T_i_* indicate increased heterogeneity in serostatus across the study area. By comparing the rank of the observed test statistic (*T_obs_*) with that from the resamples, we obtained a probability of the existence of spatial heterogeneity in the spatial distribution of seropositive samples. We considered 95% or more of all resamples *(T_i_)* with a lower test statistic than *T_obs_* as evidence to reject the null hypothesis of no spatial heterogeneity in serostatus.

#### Age distribution of pigs

The ages of all pigs (whether they were tested for JEV antibody or not) were recorded as being over or under the age of one. To obtain a parametric form of pig ages, we assumed that pig mortality followed an exponential distribution. We then identified the rate parameter that was most consistent with the observed proportion of pigs that were over and under the age of one.

#### Force of infection estimate

By assuming life-long immunity following infection and that the JE epidemic is at steady state in pigs, we calculated the average force of infection that was most consistent with the observed data [26]. We found the force of infection that best fitted the observed seroprevalence curve using a maximum likelihood method [26] (Text S1).

#### Compartmental SEIR model

To describe JEV transmission dynamics in the study region we built a compartmental SEIR model. Pigs were born into either a susceptible (S) compartment or a maternal antibodies (M) compartment; pigs in the (M) compartment later moved to the (S) compartment after a mean of four months. Susceptible pigs entered the exposed (E) compartment once they could become infected. Exposed pigs moved to the infectious (I) compartment after a mean delay of 10 days, representing the total extrinsic incubation period of the mosquito and the time from infection to viremia in the pig [27]. Infectious pigs remained in the infectious (I) compartment for a mean of 4 days [13]. Finally pigs that were no longer viremic entered the recovered (R) compartment and were considered immune to reinfection. The proportion of pigs born with maternal antibodies was determined by the proportion of pigs with a history of infection at that time point. The rate at which susceptible pigs became infected depended on the number of infectious pigs. In addition, while pigs are believed to be the critical amplifying host in these communities, infections from external hosts (e.g., wading birds) may be important. The base model assumed that five per cent of pig infections were caused by external introductions, however, as the level of external introductions is poorly understood, we varied this substantially in sensitivity analysis (see below). We fitted the model to our estimate of the force of infection by varying the rate of secondary infections per year for each infectious pig (β) in a model run to steady state using a quasi-Newton method [28]. We used the model to estimate the number of secondary infections caused by a single infectious pig in a completely susceptible pig population (the basic reproductive number, R_0_) by dividing the total number of individuals in the S, E, I and R compartments with the total number in the susceptible (S) compartment only at equilibrium. All model parameters are shown in Table 1. Details of the model and all model equations are provided in the Supplementary Information accompanying this article.

**Table 1:** Parameters used to fit model of reduction in incidence of Japanese encephalitis infection among pigs using pig vaccination.

Parameter	Value	Sources
Baseline mortality rate, per year	0.94	Fitted to observed distribution of ages
JE associated mortality	0	Model assumption
Time with persistence of maternal antibodies, months	4	[13]
Rate of secondary infections for an infectious pig, per day	0.3	Fitted to estimated force of infection (λ)
Incubation period, days	10	[13] (1)
Time infectious, days	4	[13]
Existing vaccination rate, per year	0	Model assumption
Vaccine efficacy, %	0.95	[33]
Proportion of infections that come from outside study area	0.05	Model assumption

**Notes:**

(1) Calculated as sum of 6 days extrinsic incubation period (value estimated in settings at 28°C) and 4 days incubation period within pigs.

#### Impact of vaccination

To estimate the effect on incidence from pig vaccination, we introduced a vaccinated (V) compartment. There exist a number of commercially available swine vaccines that are routinely used on commercial pig farms (e.g., manufactured by Biken in Japan and Komipharm in South Korea), however, there are very limited data available about the efficacy of these vaccines. In the base model we assumed a highly effective vaccine (95% efficacy) vaccine, however, in sensitivity analysis we considered an alternative model with reduced efficacy (50%). At each time step, a proportion of susceptible pigs without maternal antibodies were transferred to the V compartment at a defined rate reflecting the vaccination of pigs. Protection from vaccination was determined by the estimated efficacy of the vaccine. We ran four different scenarios over a period of five years, reflecting different levels of vaccination coverage: 10%, 25%, 50% or 75% of pigs vaccinated. We compared the incidence to a baseline scenario in which no vaccine was introduced.

#### Sensitivity on rate of external introductions

The proportion of pig infections that are external introductions into the pig population, meaning they originate from infections in other reservoir hosts, may impact the effect of any vaccination campaign. To estimate the potential impact of external introductions, we varied the proportion of infections that were external introductions between 0 and 10% (refitting the model in each case) and recalculated the impact on incidence.

### Ethical considerations

All pig-raisers provided informed consent for participation. The study protocol was reviewed and approved by icddr,b′s Ethical Review Committee, Animal Experimentation Ethics Committee of Bangladesh and CDC's Institutional Animal Care and Use Committee.

## Results

We identified 11,364 pigs (*Sus scrofa*) in the study area with a mean of 1.5 pigs per km^2^; the majority (61%, n = 6963) were over 12 months of age and 50% were female. Most (88%, n = 9977) of the pigs were raised in backyards (median per backyard: 1 pig, range: 1–25) with the remainder (12%; n = 1387) raised in 28 nomadic pig herds (median pigs per herd: 39, range: 6–145) (Table 2). Of the 28 herds, we mapped grazing routes of 9 herds. The pig herding routes had an average length of 108 km per year (range: 30–329 km per year). We also visited 20 (52%) of the 111 unions (administrative cluster of villages) where we did not receive any information on pig raising through pig raiser's social informational network and could only identify an additional five pigs, suggesting our approach was able to capture the vast majority of pigs.

**Table 2:** Demographics and Japanese encephalitis IgG antibody prevalence among pigs in Rajshahi, Nawabgonj, and Naogaon Districts, 2009.

	No. pigs (%)	No. pigs sampled	No. with IgG antibodies (%)
Total	11364 (100%)	312	92 (30%)
Rajshahi	3918 (34%)	100	19 (19%)
Nawabgonj	1435 (13%)	103	43 (42%)
Naogaon	6011 (53%)	109	30 (28%)
<12 months	6963 (61%)	32	5 (16%)
≥12 months	4401 (39%)	280	87 (31%)
Male	5676 (50%)	159	51 (32%)
Female	5688 (50%)	153	41 (27%)
Backyards	9977 (88%)	260	70 (27%)
Herds	1387 (12%)	52	22 (42%)

We identified 20 locations in our study area where pork and live pigs were sold. Pork was sold either daily or weekly in a pig slaughterer's backyard, which in most cases (80%) was near an established live pig market. In addition, there was one pig fair in Naogaon District held once a year during December-January. All nomadic pig herders participated in that fair to sell their pigs to backyard pig-raisers and other pig herders. Commercial pig dealers who sold pigs and pork at the markets throughout the country also participated in this fair. Other than local pig trade between the backyard farmers, the pig-raisers received the majority of their piglets from the herders. Backyard raisers mostly bought 2–3 months old piglets, which they raised for about a year or two before slaughtering for their consumption or for money. On special occasions, such as weddings and spiritual ceremonies, the backyard raiser's preferred to buy older pigs for immediate slaughter. The pig traders who sold the pigs in markets for slaughter usually bought the 5–6 month old pigs from the herders, and supplied the pork markets across the country.

We tested 312 pigs for JEV antibodies and 30% had evidence of previous infection (Table 2). Pig-raising households were primarily located in the central part of our study area (Figure 1). We failed to reject the null hypothesis of no spatial heterogeneity in the location of seropositive pigs (p-value of 0.3, Figures S1 and S2 in text S1), suggesting that the extent to which pigs were infected did not differ across the study region.

**Figure 2:**
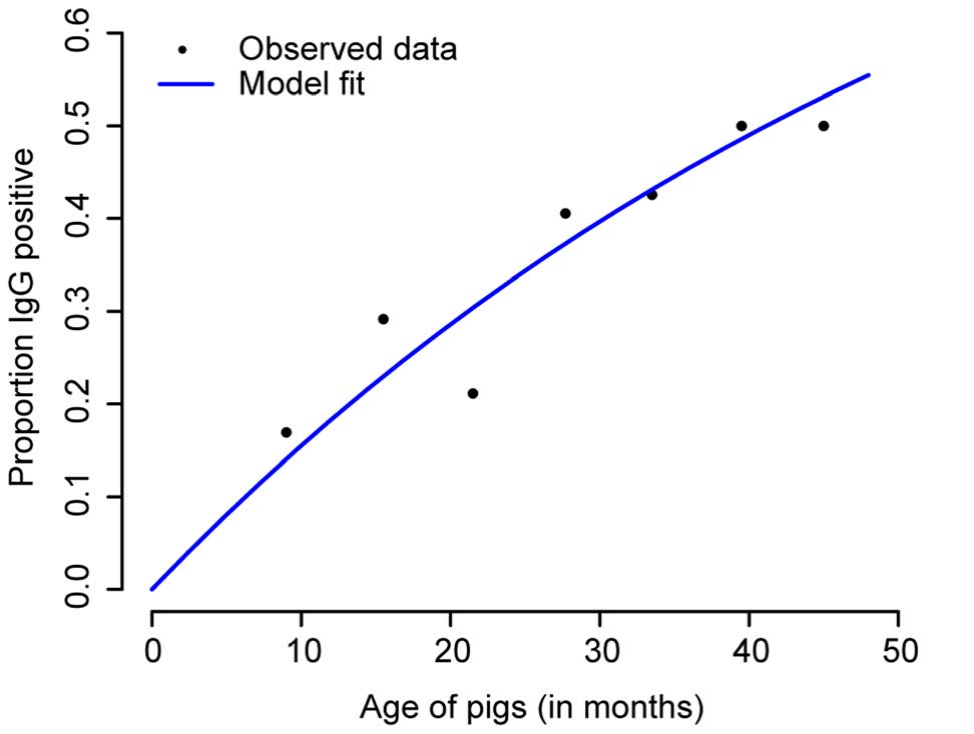
Proportion of pigs seropositive to Japanese encephalitis (JE) virus by age. 312 pigs, divided into seven age groups, were tested for presence of IgG antibodies to JE. The points are plotted at the midpoint of the age groups. The blue line represents the fit of the model assuming a constant force of infection.

Evidence of past JEV infection increased consistently with age, supporting a constant average force of infection in the pig population. Approximately 50% of pigs had evidence of past infection by 3 years of age (Figure 2). A force of infection of 0.20 per year best fitted the age distribution of seropositive cases (1/8 likelihood interval of 0.16–0.25, Figure S3 in text S1) indicating that 1 in 5 susceptible pigs got infected each year.

**Figure 3:**
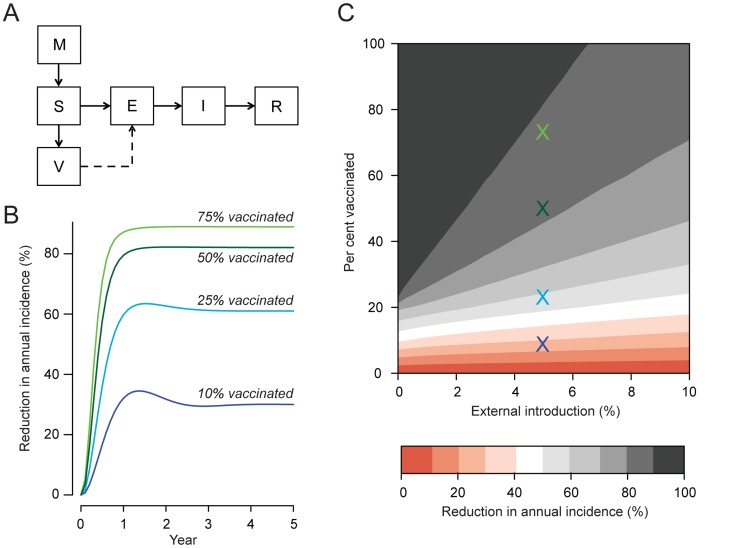
(A) Model structure indicating pigs with maternal antibodies (M), susceptible (S), exposed (E), infected (I), recovered (R), and vaccinated (V) compartments. (**B**) Estimated decrease in incidence of Japanese encephalitis (JE) in pigs under four vaccination coverage scenarios, assuming that 10%, 25%, 50% or 75% of susceptible pigs are vaccinated each year and that 5% of infections originate from outside the study area or from other hosts. (**C**) Estimated reduction in incidence of JE among pigs by proportion of the susceptible pig population vaccinated each year and proportion of all pig infections that originate from an external source. The colored crosses represent the scenarios in (**B**).

To describe the dynamics of JEV transmission in pigs we built a compartmental model and fitted it to the force of infection estimate. Using the model, we estimated that the basic reproductive ratio (R_0_) among pigs was 1.2. Therefore, on average, each infected pig would transmit to 1.2 other pigs (via mosquitoes) in a completely susceptible population. We subsequently explored the impact of vaccinating pigs. We found that vaccinating only half of all pigs each year, a potentially feasible goal from a vaccination campaign, would result in an 82% reduction in incidence, assuming that the vaccine efficacy was 95% and that 5% of pig infections result from external introductions; we would expect a small marginal benefit if we were able to vaccinate 75% of pigs (89% reduction in annual incidence) (Figure 3). In the base model, vaccinating only a quarter of pigs would result in a 61% reduction in annual incidence. We conducted sensitivity analysis that varied both the rate of external introductions and the vaccination coverage (Figure 3C). We found that even if 10% of pig infections were externally introduced, vaccinating 50% of the pigs would still result in a 72% reduction in annual incidence after five years. In a second sensitivity analyses we assumed 50% vaccine coverage, but assumed a lower vaccine efficacy of 50% (compared to 95% in the base model). Under these assumptions, vaccinating half the pigs would still result in a 54% reduction in JE incidence (compared to 82% in the base model).

## Discussion

Characterizing incidence patterns in pigs is crucial to understanding both the dynamics of JEV transmission in pig populations and the potential impact of intervention efforts. Here we conducted an intensive pig census across an area that has experienced high levels of JEV infection in humans [19]. We identified over ten thousand pigs with at least one pig found in approximately half of the unions in the study area. Evidence of past JEV infection in the pigs was found across the study area with no spatial heterogeneity in the location of seropositive pigs. There was a clear increase in seropositivity by age, characteristic of endemic diseases and we estimated that 20% of susceptible pigs get infected each year. We are not aware of previously published estimates of the basic reproductive number or the force of infection of Japanese encephalitis in pig populations.

Our model shows that by vaccinating only half (or 4,000) of the susceptible pigs each year, the incidence among pigs would be reduced by >70%, even if up to 10% of pig infections were from other animal reservoirs, such as wading birds. In other parts of Asia, pig immunization against JEV was discontinued because of logistical difficulties in keeping large herds with high annual turnovers vaccinated [29]. However, in this setting, there were fewer pigs than on large commercial pig raising operations and pigs lived for one year, on average, meaning that vaccination once per year could be sufficient to provide protection. These factors might make pig vaccination more logistically feasible in these communities. Pig-raisers in this part of Bangladesh are marginalized populations and economically disadvantaged [22] and JEV infection among sows can result in abortions and deaths of newborn pigs [30]. Although there are no published reports of using JE pig vaccination as an effective intervention to improve poverty among pig raisers, the small reduction in pig miscarriages and stillbirths from vaccination could translate into meaningful reduction in economic losses for this economically disadvantaged group. Pig raising communities are typically wary of outside intervention, and currently receive very little in the way of veterinary services provided by government-owned livestock clinics in the country [22]. Therefore, interventions to vaccine pigs could be more difficult to implement than in the setting of large commercial farms where previous vaccination efforts were attempted. Nonetheless, a pig vaccination strategy might be feasible and acceptable, particularly if it would provide some benefit to pig raisers. Pig-raising communities maintain strong relationships with one another, including through the pig trade. These close networks may provide an opportunity to efficiently access pigs and disseminate communication messages for a vaccination campaign, such as during the yearly pig-marketing event in Naogaon.

The findings from this study are subject to some important limitations. First, the number of pigs in this area may be underreported by our census if large pig-raising communities were not identified through our snowball approach. However, this appears unlikely as efforts to identify pigs in unions where no pig-raisers were reported through our snowball approach yielded very few additional pigs. Second, it is possible that we have overestimated the seroprevalence of pigs in our study sample due to cross-reactivity of antibodies to other flaviviruses [16], although there is no evidence that these viruses are endemic in pig populations in Bangladesh. A study of human encephalitis patients residing in this area found no evidence of infection with West Nile Virus (ES Gurley, personal communication), a possible viruses that often produce cross-reactions in the region [16]. Finally, we do not know the proportion of pig infections that originate from other host species. However, sensitivity analyses that varied this assumption widely still resulted in substantial reduction in pig infections from vaccination.

The high level of seropositivity in pigs in the study area suggests they may be a key reservoir for the virus and could contribute importantly to JE risk in humans. If pig infections do increase human risk substantially, reducing incidence in pigs could also result in reduced incidence in humans. All countries that have effectively reduced the burden of JE in human populations have done so through human vaccination campaigns [31]. However, there are currently no plans to introduce JE vaccine into the expanded program for immunization in Bangladesh. Given the poor prognosis for humans with JE disease and the lack of human vaccine in this area, vaccinating pigs may represent an opportunity to reduce disease risk in humans. For example, one study from Assam, India [32], which borders Bangladesh in the north, showed that human risk of JEV infection was reduced by 66% in areas where insecticide treated mosquito nets were used to prevent infections in pigs. Further studies are required to estimate the contribution of pigs in determining JE risk in humans in this area and to understand if preventing pig JEV infections could also confer a human health benefit, in the absence of human vaccination.

## Supporting Information

Text S1 Supplementary information on model parameters, equations, force of infections, kernel density bandwidth estimation, spatial heterogeneity in serostatus, and maximum likelihood estimation of the force of infection.
(DOCX)
