# Supplementary material for: Dynamics of Japanese Encephalitis Virus Transmission among Pigs in Northwest Bangladesh and the Potential Impact of Pig Vaccination
Source: PLoS Negl Trop Dis. 2014 Sep 25;8(9):e3166. doi: 10.1371/journal.pntd.0003166 (PMC4177832; doi:10.1371/journal.pntd.0003166)
Supplement: Text S1 — Supplementary information on model parameters, equations, force of infections, kernel density bandwidth estimation, spatial heterogeneity in serostatus, and maximum likelihood estimation of the force of infection. (DOCX) [file pntd.0003166.s001.docx]

Supplementary Information

# Table S1. Model parameters

| **Parameter** | **Symbol** | **Baseline** | **Source** |
| --- | --- | --- | --- |
| Mortality, per year | *µ* | 0.94 | Derived (see below) |
| Rate of maternal antibody loss, per year | *ψ* | 3 | Derived (see below) |
| Number of infections, per year | *β* | 114 | Fitted (see below) |
| Rate at which become infectious, per year | *α* | 36.5 | Derived (see below) |
| Rate of recovery, per year | *γ* | 91.25 | Derived (see below) |
| Baseline vaccination rate, per year | *υ* | 0 | Model assumption |
| Vaccine efficacy | *σ* | 0.95 | *[33]* |
| Rate of external introduction, per year | *φ* | 0.05**λ*^ˆ^ | Model assumption |

## Derived parameters

**Mortality**

Pig mortality was fitted using the data collected in the study. We found that 61% of pigs were under the age of 1. Assuming an exponential rate of mortality, this is equivalent to a mortality rate of 0.94 per year.

**Maternal antibody loss**

The rate of maternal antibody loss is calculated as the reciprocal of the mean age at which maternal antibodies wane, estimated as 4 months *[12]*.

**Rate of infectiousness**

The rate of infectiousness is calculated as the reciprocal of the mean total incubation time (both in the pig and in the mosquito), estimated as 10 days *[12]*.

**Rate of recovery**

The rate of recovery is calculated as the reciprocal of the mean time that pigs are infectious, estimated as 4 days *[33]*.

## Fitting process

We fit the model to the annual force of infection, estimated from the seroprevalence curve (Figure 2). The model was fit at steady state by optimizing the annualized number of secondary infections from each infected pig (*β*).

# Text S1. Model equations

We assumed a closed population. Rates between compartments are determined by a system of ordinary differential equations, outlined below.

## Equation 1

*dM/dt* =*µNθ*

−*ψM*

−*µM*

The first line represents births into the system and is equal to the total number of deaths from all compartments where *N* is the sum of all individuals from all compartments and *θ* is the proportion of pigs with infection history and is calculated as (*E* + *I* + *R*)*/*(*S* + *E* + *I* + *R*). The second line represents the loss of maternal antibodies and the final line is the death of pigs with maternal antibodies.

## Equation 2

*dS/dt* =*µN*(1 −*θ*)

+ *ψM*

−*µS*

−*βSI*

−*φS*

−*υS*

The first line represents pigs born without maternal antibodies. The second line represents pigs that have lost their maternal antibodies. The third line is the death of pigs. The fourth line represents pigs getting infected from infectious pigs within the study area. The fifth line is pigs getting infected from outside the study area or from non-pig hosts. The final line is the vaccination of pigs.

## Equation 3

*dE/dt* =*βSI*

+ *φS*

+ (1 −*σ*)*βIV*

+ (1 −*σ*)*φV*

−*αE*

−*µE*

The first line represents susceptible pigs that have been exposed by infectious pigs in the system. The second line is susceptible pigs that have been exposed to the virus externally. The third line is vaccinated pigs that nevertheless get infected by infectious pigs in the system. The fourth line is vaccinated pigs that get infected from external introduction. The fifth line is the development of infectiousness and the final line is pig mortality.

## Equation 4

*d***I***/dt* =*αE*

−*γI*

−*µI*

For equations 4,, the first line represents the development of infectiousness in exposed pigs. The second line is the rate of recovery of pigs. The final line is pig mortality.

## Equation 5

*dR/dt* =*γI*

−*µR*

The first line represents the recovery of infectious pigs. The second line is pig mortality.

## Equation 6

*dV/dt* =*υS*

− (1 −*σ*)*βIV*

− (1 −*σ*)*φV*

−*µV*

The first line represents the vaccination of susceptible pigs. The second line is the infection of vaccinated pigs (i.e. vaccine failure) by infectious pigs in the system. The fourth line is the infection of vaccinated pigs from outside the system and the final line is pig mortality.

**Text S3. Force of infection calculation**

We used a method by Anderson and Grenfell to calculate the mean force of infection (*λ*) [25]. Briefly, under constant force of infection and assuming that maternal antibodies have a negligible impact on the force of infection, the proportion of the individual pigs aged *a*, at time *t* that remain susceptible can be expressed as:

$$x\left( a,t \right)= exp(-\int_{0}^{a} \lambda\left( t-\tau\right)d\tau)$$

which we can estimate as:

$$\hat{x}\left( a,t \right)= exp(-\lambda a)$$

We can use a binomial likelihood to calculate the mean force of infection:

$$l\left( \lambda\right)=\sum_{k=1}^{m} [n_{k}\log\left( x\left( a,t \right) \right)+{(N}_{k}-n_{k}) log(1-x(a,t))]$$

for *m* age classes, where *N_k_* is the total number of pigs in age class *k* and *n_k_* is the number of susceptible pigs in that age class.

# Figure S1. Estimation of kernel intensity bandwidth

Estimation of the optimum Gaussian kernel bandwidth for the spatial intensity plot of the proportion of seropositive cases at any location. The dotted blue line represents the bandwidth with the lowest cross validated error.

# Figure S2. Spatial heterogeneity in serostatus

Distribution of the Diggle test statistic. The aerostats of each test was reallocated over 499 resamples with the *T_i_* test statistic calculated in each case (see methods). The grey bars represent the *T_i_* values. The black line represents the test statistic using the observed data. As over 5% of the *T_i_* values are greater than the observed test statistic, we fail to reject the null hypothesis of spatial homogeneity in aerostats (p=0.29).

# Figure S3. Maximum likelihood estimate of the force of infection

Maximum likelihood estimate of the force of infection (*λ*^ˆ^). the red line represents the 1/8 likelihood interval. The dotted black line is the maximum likelihood estimate.
